# Preconception Care Interventions for Adolescents and Young Adults to Prevent Adverse Maternal and Child Health Outcomes: Protocol for an Evidence Gap Map

**DOI:** 10.2196/56052

**Published:** 2024-05-24

**Authors:** Zahra Ali Padhani, Gizachew A Tessema, Jodie C Avery, Komal Abdul Rahim, Jacqueline A Boyle, Salima Meherali, Rehana A Salam, Zohra S Lassi

**Affiliations:** 1 School of Public Health Faculty of Health and Medical Sciences University of Adelaide Adelaide Australia; 2 Robinson Research Institute Faculty of Health and Medical Sciences University of Adelaide Adelaide Australia; 3 Curtin School of Population Health Faculty of Health Sciences Curtin University Perth Australia; 4 Centre of Excellence in Trauma and Emergencies (CETE) Aga Khan University Hospital Karachi Pakistan; 5 Dean’s Office Medical College Aga Khan University Hospital Karachi Pakistan; 6 Health Systems and Equity Eastern Health Clinical School Monash University Melbourne Australia; 7 College of Health Sciences Faculty of Nursing University of Alberta Edmonton Australia; 8 Centre of Research Excellence Melanoma Institute Australia University of Sydney Sydney Australia

**Keywords:** preconception health, adolescent, young adult, maternal health, perinatal outcomes, child health, infant health, evidence gap map, EGM, interventions, perinatal health, preconception, young woman, woman, evidence gap, perinatal, map, gap, offspring, maternal, infancy, prepregnancy

## Abstract

**Background:**

Preconception is the period before a young woman or woman conceives, which draws attention to understanding how her health condition and certain risk factors affect her and her baby’s health once she becomes pregnant. Adolescence and youth represent a life-course continuum between childhood and adulthood, in which the prepregnancy phase lacks sufficient research.

**Objective:**

The aim of the study is to identify, map, and describe existing empirical evidence on preconception interventions that enhance health outcomes for adolescents, young adults, and their offspring.

**Methods:**

We will conduct an evidence gap map (EGM) activity following the Campbell guidelines by populating searches identified from electronic databases such as MEDLINE, Embase, CINAHL, and Cochrane Library. We will include interventional studies and reviews of interventional studies that report the impact of preconception interventions for adolescents and young adults (aged 10 to 25 years) on adverse maternal, perinatal, and child health outcomes. All studies will undergo title or abstract and full-text screening on Covidence software (Veritas Health Innovation). All included studies will be coded using the Evidence for Policy and Practice Information (EPPI) Reviewer software (EPPI Centre, UCL Social Research Institute, University College London). Cochrane Risk of Bias tool 2.0 and Assessing the Methodological Quality of Systematic Reviews-2 (AMSTAR-2) tool will be used to assess the quality of the included trials and reviews. A 2D graphical EGM will be developed using the EPPI Mapper software (version 2.2.4; EPPI Centre, UCL Social Research Institute, University College London).

**Results:**

This EGM exercise began in July 2023. Through electronic search, 131,031 publications were identified after deduplication, and after the full-text screening, 18 studies (124 papers) were included in the review. We plan to submit the paper to a peer-reviewed journal once it is finalized, with an expected completion date in May 2024.

**Conclusions:**

This study will facilitate the prioritization of future research and allocation of funding while also suggesting interventions that may improve maternal, perinatal, and child health outcomes.

**International Registered Report Identifier (IRRID):**

DERR1-10.2196/56052

## Introduction

Preconception health influences the reproductive, perinatal, and child health outcomes of women who are planning their pregnancy [1]. According to the United Nations Population Fund, nearly half of all pregnancies worldwide are unplanned [2,3]; thus, risk factors that can potentially lead to adverse perinatal, maternal, and child outcomes are identified and addressed in the initial prenatal appointment, which usually marks the midpoint of the critical first trimester [4].

Existing literature focuses on identifying risk factors and interventions to prevent adverse maternal, perinatal, and child outcomes among women of reproductive age, who are either pregnant or in their postpartum period [5,6]. However, exposures occur a relatively long time before conception (during adolescence), which has indeed received very limited attention [7]. Adolescence, a phase from childhood to adulthood (from ages 10 to 19 years), marks a critical period of growth and development physically, socially, psychologically, and intellectually. This period also includes the social preparation for marriage or partnering and maturity before reaching adulthood [8]. Since adolescents have fully developed their life skills, they are at greater risk of indulging in risky behaviors such as substance abuse and unsafe sex, leading to adverse outcomes such as unintended pregnancies, pregnancy complications, sexually transmitted diseases, preterm birth, low birth weight (LBW), congenital anomalies, intrauterine growth restriction, behavioral problems, and infant and maternal mortality [9-11].

Preconception care focuses on identifying health problems, poor lifestyle habits, and social health before, during, and after pregnancy, as well as during adolescence [12]. Preconception health interventions can modify behavioral, biomedical, and social risks pertinent to women’s health via prevention and management strategies [13]. It increases the need for optimal health before conceiving a baby [14]. Preconception care is a broader umbrella comprising several components such as risk assessment or screening, health promotion and counseling, interventions targeting the identified risks, and referral to specialized preconception care if indicated [15-17].

Screening and risk assessment as part of preconception care helps to assess and gauge a holistic picture of the risk factors that can adversely affect perinatal outcomes [18]. Evidence suggests that perinatal screening and risk assessment help identify the trend of perinatal mortality [19], congenital anomalies [20,21], and general health [21]. Family planning, psychosocial counseling, empowerment of women, health promotion, and education are also important components of preconception care, which aim to increase awareness and modify an individual’s behavior, preventing risky behaviors such as substance abuse and unsafe sex [7]. It can also help reduce the risk of unintended pregnancies [22], unsafe abortions [23], sexually transmitted infections, maternal morbidity, and mortality [24]. Another important aspect of psychosocial counseling and health promotion is around healthy weight and nutritional supplementation [25]. Interventions targeting poor outcomes due to inadequate nutrition include social protection programs, food fortification, nutrition counseling, lifestyle modification, and both macro- and micronutrient supplementation [7,25-27]. For example, folic acid supplementation reduces the risk of neural tube defects [28], and iron supplementation reduces the risk of maternal anemia [29]. Multiple micronutrient supplementation in pregnancy has also been shown to reduce the likelihood of a baby who is LBW [27,30], is small for gestation age (SGA) [31], and is premature [27,32]. Social support and social protection programs, such as safe spaces for adolescents and young people, food baskets, conditional cash transfers, and food vouchers, have contributed to improving the mental health and health and nutrition of adolescents, mothers, and their families [33-35]. These programs have also contributed to alleviating issues faced due to poverty and low socioeconomic status and have helped reduce the risk of infant mortality, preterm, LBW, and SGA babies [33]. These interventions have also contributed to reducing the risk of psychosocial stressors, incidence of domestic and sexual abuse, female genital mutilation, and substance abuse among adolescents [34,36].

Despite all the identified interventions in preconception care, the target of positive health outcomes in adolescents and their children has not yet been achieved. Given that many exposures can lead to adverse outcomes in pregnant adolescents and developing fetuses, targeted interventions to improve overall health are understudied. Therefore, we aim to identify, map, and describe the gaps in the existing empirical evidence on preconception health interventions implemented to improve the health and well-being of adolescents and young adults (10 to 25 years of age) and their children.

## Methods

### Overview

We will undertake an evidence gap map (EGM) exercise to identify the gaps in existing evidence on preconception health interventions for adolescents and young adults. EGM analysis visually presents the existing evidence, presenting different types of interventions and outcomes evaluated. EGM is an intuitive tool widely used for identifying gaps in evidence and for decision-making of policy [37]. For the EGM exercise, Campbell’s checklist will be followed for reporting standards of the EGM [38,39], and the PRISMA (Preferred Reporting Items for Systematic Reviews and Meta-Analyses) guidelines will also be followed for reporting the purposes [40]. The EGM is also registered at Open Science Framework [41].

### Eligibility Criteria

The eligibility criteria for the EGM exercise have been briefly discussed below.

#### Topic of Interest

In this EGM, studies that have reported on preconception health interventions among adolescents and young adults to prevent adverse maternal and child health outcomes will be included.

#### Population

We will include studies involving nonpregnant adolescents and young adults aged 10 to 25 years (of all genders) at the time of intervention. We will exclude studies conducted on women of reproductive age older than 25 years; however, we will include studies that report subgroup or disaggregated data on adolescents and young adults. Additionally, we will consider studies on a case-by-case basis if the majority of participants were adolescents and young adults, as determined by assessing the median age at the time of intervention.

#### Study Design

Systematic reviews of interventions will be included, but for systematic reviews including both interventional and observational studies, data will be extracted from the evidence coming from interventional studies included in the review.

We will also include experimental studies (such as individual or cluster randomized and nonrandomized controlled trials, including quasi-randomized, controlled before-after, and interruptive time series) focused on preconception interventions. Observational studies, qualitative studies, descriptive reviews, narrative reviews, literature reviews, and gray literature will be excluded.

Initially, we will search for systematic reviews on the topic. A separate search will be conducted for experimental studies that did not have a published systematic review on the topic.

#### Intervention

We will include studies reporting on any intervention intended to improve preconception health compared to any other intervention, standard of care, or no intervention. Previously published reviews and reports served as a guiding road map for preconception intervention [13,42,43]. These interventions include general preconception care, health promotion, sex education, family planning services (including counseling, contraceptive distribution, and birth intervals), nutrition counseling and supplementation, social protection programs, lifestyle modification, vaccination or immunization, screening and management of chronic diseases, prevention of domestic violence, genetic monitoring and testing, social support, telemedicine, vocational training for youth development and empowerment, smoking cessation, prevention of alcohol and substance use (including drug therapy, rehabilitation, and psychosocial support), dental care and hygiene practices, prevention from chemical and environment risk, mental health programs, and prevention and treatment of infections (including sexually transmitted infections). We will exclude studies on infertility treatment.

#### Outcomes of Interest

We will only include studies that have reported on the impact of preconception health interventions on maternal, perinatal, and child health outcomes, regardless of whether the assessments occurred during adolescence or at any subsequent point following the intervention. We will exclude studies that have not reported on the following outcomes: (1) maternal outcomes include maternal morbidity (as reported by the authors but mainly include outcomes like anemia, gestational weight gain, pre-eclampsia, and postpartum hemorrhage), maternal mortality, pregnancy complications, and mode of delivery (vaginal or cesarean section); (2) perinatal outcomes include miscarriage or spontaneous abortions, stillbirth, perinatal mortality, neonatal mortality, preterm birth, SGA or large for gestation age, intrauterine growth retardation, LBW (<2500 g) or macrosomia (>4000 g), mean birth weight, and congenital anomalies or birth defects; and (3) child and infant outcomes include infant mortality (death under 1 year), child mortality (death under 5 years), early childhood morbidity (as reported by the authors but mainly include outcomes like fever, diarrhea, pneumonia, and other infections), poor growth outcomes, developmental outcomes (eg, poor motor skills), sexually transmitted diseases (transferred through mothers, eg, HIV or AIDS), and mental health issues or disorders

### Search Strategy and Databases Search

A search strategy was developed using keywords and MeSH terms on preconception health interventions, adolescents, young adults, and maternal, perinatal, and child health outcomes (Multimedia Appendix 1). The following databases will be used to search the relevant papers: CENTRAL, MEDLINE, Embase, CINAHL, PsycINFO, and Web of Science. All the systematic reviews will be cross-referenced to retrieve studies that would have been missed during the initial search. The searches will be limited to language and date, that is, studies published in English from 2010 onward. We plan to search data from 2010 because the World Health Organization started a momentum to improve preconception health and address poor maternal and perinatal outcomes through stakeholder meetings across different regions worldwide [13,44]. Through this meeting, the World Health Organization addressed the need for a continuum of care from preconception through pregnancy, aiming to reduce maternal and childhood mortalities by advocating the integration of preconception care into global health strategies.

### Screening and Data Extraction

All studies identified from the search will be imported to the Covidence software (Veritas Health Innovation), and 2 independent reviewers will screen the studies at the title or abstract and full-text stages, after removing the duplicate studies. The senior reviewer (ZSL) will resolve any disagreements. The final list of included studies will be subject to forward and backward citation chaining to ensure no relevant eligible papers have been missed. A diagram showing the flow of literature will be produced and reported in accordance with PRISMA guidelines [45]. Data will also be extracted in the framework matrix, and mapping will be done independently. Data will be extracted on study design, participants, study setting, intervention, comparison groups, and the outcome assessed.

### Quality Assessment of Included Studies

We will appraise the quality of systematic reviews using Assessing the Methodological Quality of Systematic Reviews-2 (AMSTAR-2) [46] and Cochrane Risk of Bias 2.0 tool for randomized controlled trials and cluster randomized controlled trials [47], and Risk of Bias in Non-Randomized Studies of Interventions (ROBINS-I) for quasi-experimental studies [48]. Two independent reviewers will assess the quality of the included studies, and discrepancies will be resolved through discussion or by contacting the third reviewer (ZSL). The risk of bias assessment of the included studies will highlight the quality of the included trials and reviews, which will help researchers and policy makers to plan studies and programs strategically in the future.

### Analysis Approach

**Figure 1:**
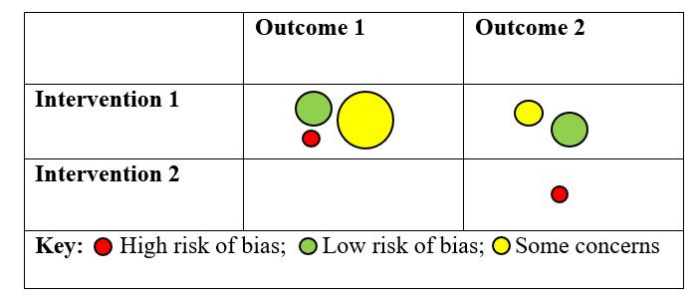
A visual example of an evidence gap map.

The EGM will be created on Evidence for Policy and Practice Information (EPPI) Reviewer (EPPI Centre, UCL Social Research Institute, University College London) and EPPI Mapper software (version 2.2.4; EPPI Centre, UCL Social Research Institute, University College London), where we will use the 2D intervention outcome framework to provide a visual representation of evidence gaps in the literature related to the interventions implemented to improve preconception health. The 2D EGM framework will be formed following the eligibility criteria, in which the interventions would be set as rows and outcomes as columns, and the bubble in the cells of the table will show the studies reporting on that particular intervention and outcome. Empty cells in the table will highlight no evidence on that particular intervention and outcome. The size of the bubble will signify the number of studies, while the color of the bubble will show the quality of the study, gender, or any other selected variable. The maps will be populated based on quality assessment of included studies, gender, age of participants at the time of intervention (adolescents: 10 to 18 years, young adults: 19 to 25 years, and both adolescents and young adults), and country by income. A visual example of EGM is given in Figure 1. The key findings from the EGM will be reported narratively.

### Ethical Considerations

Ethics approval is not required, as this paper is an EGM of publicly available and published literature.

## Results

This EGM exercise began in July 2023. Through electronic search, 131,031 publications were identified after deduplication, and after the full-text screening, 18 studies (124 papers) were included in the review. Data analysis and summarization are currently under process. We plan to submit the paper to a peer-reviewed journal once it is finalized, with an expected completion date in May 2024. The findings of this study will identify and highlight the research gaps in preconception health interventions in different contexts and geographical settings. The results of the study will be disseminated among researchers and other stakeholders through publication in a peer-reviewed journal and presentation at national and international conferences. Additionally, we will promote the study’s findings on various social media platforms, including Twitter and LinkedIn, and we will also write blogs and newspaper articles to share the results with a broader audience.

## Discussion

### Overview

To the best of our knowledge, this is the first EGM that will collate all the existing evidence on preconception health interventions targeted to adolescents and young adults to prevent adverse maternal, perinatal, and child health outcomes. The 2D matrix of interventions and outcomes will help identify gaps in evidence and will inform policy and funding agencies for further specific areas of research.

### Strengths and Limitations

The limitation of this EGM is that it will include studies published after the year 2010 and those published in the English language, thus leading to a loss of studies published prior to 2010 and those published in other languages. The strength of this study is that we will follow the Campbell checklist to report the study findings. We will also follow a systematic approach to search papers, which also involves dual screening and data extraction process. We will also conduct a risk of bias assessment to judge the quality of included trials and systematic reviews.

### Conclusions

Adolescents and young adults are a part of an underresearched population. It is also a global health priority to identify, target, and engage adolescents and young adults at preconception in healthy lifestyle interventions to improve health outcomes. The EGM will help map out the spread of preconception care interventions and programs specifically planned and carried out for adolescents and young adults. It will also highlight the evidence on effective preconception interventions for adolescents and young people to improve their health and the health of their future generation and will identify the research gaps in the current evidence base to point out areas in preconception care that underscore research attention for further work. The proposed project is the first step toward identifying interventions that have health and economic benefits for the larger younger population.

## Appendix

Multimedia Appendix 1 Search strategy for MEDLINE.

## Abbreviations

AMSTAR-2: Assessing the Methodological Quality of Systematic Reviews-2
EGM: evidence gap map
EPPI: Evidence for Policy and Practice Information
LBW: low birth weight
PRISMA: Preferred Reporting Items for Systematic Reviews and Meta-Analyses
ROBINS-I: Risk of Bias in Non-Randomized Studies of Interventions
SGA: small for gestation age
