# Supplementary material for: Preconception Care Interventions for Adolescents and Young Adults to Prevent Adverse Maternal and Child Health Outcomes: Protocol for an Evidence Gap Map
Source: JMIR Res Protoc. 2024 May 24;13:e56052. doi: 10.2196/56052 (PMC11161710; doi:10.2196/56052)
Supplement: Multimedia Appendix 1 [file resprot_v13i1e56052_app1.doc]

1 exp Adolescent/ or exp *Adolescent Health/ or exp Young Adult/ or (preconception health or pre conception health or pre pregnancy health or prepregnancy health or before pregnancy or pre conception or preconception or prepregnancy or preconceptional or periconceptional or before conception or prior conception or young adult* or young women or young woman or young men or young man or young people or young* or youth* or teen* or young people or young person* or adolesc* or preadolesc* or pre-adolesc* or juvenil* or minor* or p?ediatric* or pubescen* or pre-pubescen* or prepubescen* or puberty or high-school* or highschool*).ti,ab.

2 exp Preconception Care/ or exp *Adolescent Health Services/ or exp Reproductive Health Services/ or exp Family Planning Services/ or exp Contraception/ or exp Prenatal Care/ or exp Ambulatory Care Facilities/ or exp Safe Sex/ or exp Sex Education/ or exp Maternal Health Services/ or exp Telemedicine/ or exp Community Participation/ or exp Empowerment/ or exp Health Promotion/ or exp Health Education/ or exp Birth Intervals/ or exp Early Intervention, Educational/ or *Condoms/ or *Condoms, Female/ or *Contraceptive Devices, Male/ or *Contraceptives, Oral/ or *Intrauterine Devices/ or *Antiretroviral Therapy, Highly Active/ or exp Nutrition Assessment/ or exp Diet/ or exp Exercise/ or *Counseling/ or *Awareness/ or *Home Care Services/ or public assistance/ or food assistance/ or medical assistance/ or social security/ or exp Iron, Dietary/ or exp Dietary Supplements/ or exp Micronutrients/ or exp Folic Acid/ or exp Vitamins/ or *Minerals/ or *Dietary Proteins/ or exp Healthcare Financing/ or exp Obesity Management/ or exp School Health Services/ or *Curriculum/ or exp Food, Fortified/ or exp Behavior Therapy/ or exp Immunization/ or exp Vaccination/ or exp Immunization Programs/ or exp Preventive Health Services/ or exp Psychosocial Intervention/ or exp Motivation/ or Rehabilitation, Vocational/ or Psychiatric Rehabilitation/ or exp Rehabilitation/ or exp Employment, Supported/ or exp Smoking Prevention/ or exp Smoking Cessation/ or exp Tobacco Control/ or *Drug Therapy/ or exp Anthelmintics/ or exp Genetic Testing/ or exp Genetic Counseling/ or *Environmental Monitoring/ or *Biological Monitoring/ or exp Disease Management/ or exp Dental Care/ or exp Oral Hygiene/ or (intervention or pre conception care or pre pregnancy care or prepregnancy care or perinatal care or post abortion care or family planning or contraceptive* or Youth friendly family service* or Digital media technology or service* or participatory learning or empower* or campaign* or emergency contraception or delay* pregnancy or inter pregnancy interval or inter-pregnancy interval or birth spacing or early childhood education or adolescent-friendly place* or adolescent-friendly space* or birth control or education or counsel* or community mobilization or health visit* or home visit* or screening or physical activity or IFA supplement* or supplement* or folate or iron or zinc or calcium or iodine or multivitamin* or micronutrient* or prenatal vitamin* or energy protein or food basket* or life style modification or lifestyle modification or metabolic control or cash transfer* or food voucher* or conditional cash transfer* or CCT or cash voucher* or insurance* or UCT or unconditional cash transfer* or microcredit or incentive* or healthcare financ* or health care financ* or Program* or health program* or obesity prevention or manage* or prevent* or treat* or social protection or Life-skills curriculum or social support or nutrition counsel* or nutrition optimi?ation or balanced diet or food fortification or sanitation or hygiene or vaccin* or vaccine* or immunisation or couple counsel* or group counsel* or relationship building or vocational train* or psychosocial care or therap* or treatment* or medication* or psychological intervention* or music therap* or taxation or genetic screen* or genetic consultation or bio-monitoring or biomonitoring or monitor* or chronic care or chronic disease manage* or dental hygiene or mouth hygiene or tooth hygiene or teeth hygiene or oral care).ti,ab.

3 exp Pregnancy Outcome/ or exp Obstetric Labor Complications/ or exp Abortion, Spontaneous/ or exp Maternal Mortality/ or exp Sexually Transmitted Diseases/ or *Pre-Eclampsia/ or *Delivery, Obstetric/ or exp Pregnancy Complications/ or *Postpartum Hemorrhage/ or *Cesarean Section/ or *Obstetric Labor Complications/ or *Uterine Rupture/ or *Cesarean Section/ or exp Diabetes, Gestational/ or *Venous Thromboembolism/ or *Thromboembolism/ or exp Stillbirth/ or exp Premature Birth/ or exp Perinatal Mortality/ or *Mortality/ or exp Morbidity/ or exp Child Mortality/ or exp Fetal Mortality/ or exp Infant Mortality/ or Infant, Low Birth Weight/ or *Asphyxia/ or exp Infant, Small for Gestational Age/ or exp Birth Weight/ or exp Fetal Growth Retardation/ or *Fetal Macrosomia/ or *Dwarfism/ or exp Growth Disorders/ or exp Abnormalities, Multiple/ or exp Congenital Abnormalities/ or exp Limb Deformities, Congenital/ or *Neural Tube Defects/ or exp Chronic Disease/ or exp Anemia/ or *Attention Deficit Disorder with Hyperactivity/ or exp Mental Disorders/ or exp Noncommunicable Diseases/ or exp Intellectual Disability/ or exp Motor Skills Disorders/ or exp Cognitive Dysfunction/ or exp Pregnancy Complications, Infectious/ or exp Developmental Disabilities/ or exp Prenatal Exposure Delayed Effects/ or exp Neurodevelopmental Disorders/ or exp Child Development/ or exp Child Behavior Disorders/ or *Wasting Syndrome/ or *Wasting Disease, Chronic/ or *Obesity/ or *Overweight/ or *Diabetes Mellitus/ or exp *Spinal Dysraphism/ or exp Heart Defects, Congenital/ or *Gastroschisis/ or *Phenylketonurias/ or *beta-Thalassemia/ or *Thalassemia/ or *Neoplasms/ or *Anemia, Sickle Cell/ or *Hematologic Diseases/ or *Asphyxia Neonatorum/ or *Clubfoot/ or *Pregnancy, Ectopic/ or *Placenta Previa/ or *Abruptio Placentae/ or *Autistic Disorder/ or *Down Syndrome/ or *Anencephaly/ or exp Urogenital Abnormalities/ or exp Digestive System Abnormalities/ or exp Nervous System Malformations/ or *Facial Asymmetry/ or *Cleft Lip/ or *Chromosome Aberrations/ or exp Musculoskeletal Abnormalities/ or *Diabetes Mellitus, Type 1/ or exp Fetal Distress/ or exp Mental Health/ or (death* or neonatal death* or neonatal mortalit* or fetus mortality or prenatal mortality or premature mortality or preterm birth* or miscarriage* or anaemia or foetal death* or cancer* or obstructed labour or large for gestational age or intrauterine growth retardation or congenital anomal* or limb reduction defect* or restricted growth or birth defect* or maternal infection* or infection* or C-section or mental retardation or congenital malformation* or stunt* or spina bifida or neonatal hypoxia or chronic disease* or blood disorder* or perinatal asphyxia or intrauterine death or placental abruption or facial deformit* or active phase arrest).ti,ab.

4 exp non-randomized controlled trials as topic/ or exp randomized controlled trials as topic/ or (trial* or randomised controlled trial* or RCTs or cRCTs or cluster randomised or cluster randomized or individually randomised or individually randomized or quasi randomised controlled trial* or quasi randomized controlled trial* or controlled before-after or controlled before after or interruptive time series or non-randomised controlled trial* or program evaluation or experimental or interventional study or control trial*).ti,ab.

5 1 and 2 and 3 and 4

6 exp animals/ not (exp animals/ and exp humans/)

7 5 not 6

8 limit 7 to (english language and yr="2010 -Current")
